# BUILDING SUSTAINABILITY IN PUBLIC HEALTH ALZHEIMER’S DISEASE PROGRAMS

**DOI:** 10.1093/geroni/igad104.0852

**Published:** 2023-12-21

**Authors:** Lisa McGuire, Heidi Holt

**Affiliations:** CDC, Atlanta, Georgia, United States; Centers for Disease Control and Prevention, Atlanta, Georgia, United States

## Abstract

While public health experts cannot yet say how to prevent Alzheimer’s disease and related dementias (ADRD), public health has a role in slowing ADRD through the implementation of risk reduction strategies, early diagnosis, and better education and training of front-line health care professionals. Efforts to enhance the sustainability of programs, with changes to systems, environments, and policies enhances uptake and increases the lifespan of these interventions, as well as facilitates continuity and integration of efforts across jurisdictions. This approach includes multiple partners from a wide-range of sectors. This presentation will describe public health approach to ADRD including increasing awareness and action on dementia risk reduction, early detection, and coordination with aging services including supporting dementia caregivers. We will discuss innovative approaches of achieving sustainability, including identifying approaches that seek to reach entire populations for widespread and sustained impact utilizing examples from BOLD Programs to develop widespread public health infrastructure.

